# Periprosthetic Joint Infections: Clinical and Bench Research

**DOI:** 10.1155/2013/549091

**Published:** 2013-10-27

**Authors:** Laurence Legout, Eric Senneville

**Affiliations:** ^1^Infectious Disease Department, Alpes-Leman Hospital, Route de Findrol, 74130 Contamines sur Arve, France; ^2^Infectious Disease Department, Dron Hospital, Rue du President Coty, 59208 Tourcoing, France

## Abstract

Prosthetic joint infection is a devastating complication with high morbidity and substantial cost. The incidence is low but probably underestimated. Despite a significant basic and clinical research in this field, many questions concerning the definition of prosthetic infection as well the diagnosis and the management of these infections remained unanswered. We review the current literature about the new diagnostic methods, the management and the prevention of prosthetic joint infections.

## 1. Introduction

Prosthetic joint infection (PJI) causes significant morbidity and accounts for a substantial proportion of heath care expenditures in hospital. By the use of adapted perioperative antimicrobial prophylaxis, laminar airflow surgical environment has reduced the risk of infection to less than one percent for hip prosthesis and 2 percent for other prostheses [1, 2]. These rates are probably underestimated because of unrecognized infection. Uniform criteria are not well defined for PJI and are based on clinical, biological criteria and radiological findings. The identification of the pathogen(s) involved in the infectious process is crucial in order to maximize the chances of cure and is based on microbiological examination of synovial fluid and peroperative samples. With newer diagnostic techniques, such as sonication of removed implants, molecular methods, and mass spectrometry, the sensitivity of diagnostic methods has been significantly increased [3–6]. The key of the management of PJI is the removal of infected prosthesis, although recent studies suggest that the retention of the infected implants may be an acceptable option in selected patients. The choice of antimicrobial regimens is based on the results established from experimental studies and clinical experiences. Randomized studies are lacking [1, 2, 7–9].

This review aims to provide updated data on pathogenesis, diagnosis, and treatment of PJIs with a special focus on new diagnostic methods and new anti-methicillin-resistant staphylococci agents. We compared the European recommendations to recent IDSA (infectious diseases society of America) guidelines on the management of prosthetic joint infections [2, 7, 10], and we review the current knowledge on the management of resistant bacterial, fungal, and mycobacterial infections.

## 2. Pathogenesis

### 2.1. Biofilm

PJIs are typically caused by microorganisms organized in structured colony, surrounded by an extracellular matrix produced by these bacteria that adhere on an inert support. The special conditions of life existing within the biofilm generate phenotypic changes of the microorganisms. The bacteria living in a biofilm usually harbor resistant to most antibiotics by combination of several mechanisms including target modification, efflux, and secretion of inactivating enzymes, as a result of the alterations in the bacterial metabolism. All these events, reducing the effectiveness of antibiotics, increase the risk of chronicity and recurrence. It is also believed that chronicity and recurrence are related at least in part to the existence of microbiological cells in a stationary growth phase [1, 10, 11]. Adhesion of bacteria is due to a primary attachment mediated by specific adhesins and by nonspecific factors. The factors influencing bacteria adherence to the prosthesis surface include chemical composition, surface charge, hydrophobicity, and simply surface roughness or physical configuration of biomaterials [10, 12–14].

## 3. Surface Treatment for Bacterial Adhesion in Newly Designed Implants

The characteristics of implants such as surface roughness, chemistry can be modified to counteract bacterial colonization. For example, ultraviolet light radiation can lead to an increase in spontaneous wettability on titanium dioxide, which can inhibit bacterial adhesion [15, 16]. In addition to physiochemical modifications on the biomaterial surface, polymer coatings can be applied on the surface of titanium implants [17, 18]. Recently, Holinka et al. [18] have evaluated the bacterial adhesion of *S. aureus* and *S. epidermidis* with titanium disk who were coated with increasing concentrations of selenium. They demonstrated that both strains showed a significantly reduced attachment on titanium disk with 0.5% and 0.2% selenium concentrations. Silver impregnated coating is another attractive option [19, 20]. However, further information is needed regarding its long term tissue toxicity and the potential acquisition of resistance. Other coatings have been studied (organic agents such as chlorhexidine that is released gradually over several days, some bioactive molecules such as hyaluronic acid which has the ability to prevent bacterial adhesion), but there is still insufficient in vivo evidence indicating that these substances support osteointegration compared with other coatings like calcium phosphate [17]. Recently, the concept of multiple functionalities for surface coating of implants has been explored [17, 20, 21], but this approach is still in development.

## 4. Clinical Presentation

Prosthetic joint infections are classified as “early” (those occurring within 3 months of implantation), “delayed” (3–12 months after implantation), and “late” (more than 12 months after implantation). Early and delayed infections are thought to be due to organisms introduced at the time of surgery, whereas late infections are more likely to be hematogenously acquired. The most frequent source of bacteremia is skin, respiratory, dental, and urinary tract infections [1]. Early infections typically present as an acute joint pain, effusion, erythema at the site of implantation, and fever. During the course of infection, sinus tract with purulent discharge may occur. Patients with delayed PJI usually present more latent signs as unusual pain, implant loosening, or both. Sometimes, the clinical presentation is only a small sinus tract that opens and closes in the course of infection. The risk of PJI is highest during the first two years after implantation but persists at lower levels as long as the prosthesis remains in place. Three studies that evaluated the risk of bacterial seeding on a prosthetic joint after staphylococcal bacteremia reported an incidence of 29–40% [22–24].

## 5. Diagnosis of Prosthesis Joint Infection

The diagnosis of infection is evoked on a combination of clinical, histological and biopsy, or intraoperative microbiological criteria. However, there is no uniform criteria for the definition of PJI.

## 6. Laboratory Studies

Routine blood tests, especially elevated CRP (C-reactive protein) and/or leukocyte count, may suggest a diagnosis of infection (but are unhelpful in the early postoperative phase as they will be raised for around 14 days after surgery). However, persistent elevation of CRP raises the possibility of infection. A low CRP may help rule out infection. Fink et al. [25] reported that a CRP of less than 13.5 mg/L had a negative predictive value of 88.5% in the diagnosis of late prosthetic knee infection. An elevated CRP had a positive predictive value of only 59.2%. It should be emphasized that normal results do not exclude infection and abnormal results may reflect pathology elsewhere. The erythrocyte sedimentation rate is not enough to rule out PJI because this parameter can be influenced by protein or hemoglobin rate. Leukocyte count and procalcitonin blood levels have low sensitivity for detecting PJI [26, 27].

## 7. Microbiology

### 7.1. Conventional Diagnostic Methods

The diagnosis and determination of etiology of PJI depend on the isolation of the microorganisms from reliable samples like blood cultures of blood or intraoperative specimens or joint aspiration [7, 25, 28–30]. In case of concomitant cellulitis, aspiration should be performed through noninfected skin area. Culture of superficial wound or sinus tract infections should be avoided because they usually reflect the microbial colonization from the surrounding skin. Culture specimens should be obtained before antibiotics are initiated. All experts agree to accept a minimum of three intraoperative tissue specimens for culture [1, 7, 31]. To detect cases of low-grade infection, antimicrobial therapy should be discontinued for a minimum of 2 or 3 weeks before tissue specimens are obtained. Administration of preoperative antibiotics is acceptable in rare cases where severe sepsis requires immediate antibiotic therapy. Histology is time consuming and does not indicate which bacteria is responsible for PJI nor provides data on the susceptibility profile of the strains. However, histology can help the physician to prove the inflammatory reaction and sometimes to find other diagnoses such as cancer.

Synovial-fluid aspiration and differential cells count are useful in the preoperative diagnosis of PJI [29, 30]. Trampuz et al. [30] reported that a synovial-fluid leukocyte count of more than 1.7 × 10^3^/mm^3^ or a differential count with more than 65% of neutrophils was consistent with knee PJI. A synovial-fluid leukocyte count of more than 4.2 × 10^3^ per cubic millimeter or more than 80% neutrophils is consistent with prosthetic hip infection. Synovial-fluid culture has a sensitivity of 56 to 75% and a specificity of 95 to 100%, and to achieve optimal sensitivity and specificity, it should be performed by means of inoculation into a blood-culture bottle.

Gram's staining is not recommended in routine because of its poor sensitivity and specificity. Conventional microbiological techniques are usually used for the diagnosis of PJI. It is recommended that microbiology specimens are cultured for at least 10 days [7, 31]. Certain organisms, such as *Propionibacterium* spp. and *Corynebacterium* spp., however, may require longer incubations. Schäfer et al. [32] demonstrated that only 73.6% of infections were detected by 7 days of culture, the remainder were detected during the second week of culture.

Periprosthetic-tissue cultures can, however, be falsely negative because of previous antimicrobial therapy, low inoculum of microorganism, low number of tissue specimens, inappropriate culture medium, inadequate culture incubation time, or a prolonged time to transport the specimen to the laboratory.

### 7.2. New Diagnostic Methods

Rapid and accurate diagnostic tools which could detect a broad range of causing microorganisms and their antimicrobial resistance are increasingly needed [4, 5, 33–35].

#### 7.2.1. Sonication

Sonication of a removed implant may increase the culture yield by disrupting adherent bacteria from the biofilm. Removed orthopedic implants are sonicated in saline solution to dislodge microorganisms from the surface, followed by culture of sonication fluid.

Trampuz et al. [5] reported a sensitivity of sonicated-fluid culture superior to the standard culture of periprosthetic tissue (75% versus 54%), whereas the specificity was 87% and 98%, respectively. Sonication in bags lacked specificity due to bag leakage and contamination in this study. In a prospective trial performed by the same team [36], comparing cultures of samples obtained by sonication of explanted hip and knee prostheses with conventional culture, they obtained better result of sensitivity. With the use of standardized nonmicrobiologic criteria to define prosthetic joint infection, the sensitivities of periprosthetic-tissue and sonicated-fluid cultures were 60.8% and 78.5% (*P* < 0.001), respectively, and the specificities were 99.2% and 98.8%, respectively. Fourteen cases of prosthetic joint infection were detected by sonicated-fluid culture but not by standard prosthetic-tissue culture. In patients receiving antimicrobial therapy within 14 days before surgery, the sensitivities of periprosthetic-tissue and sonicated-fluid cultures were significantly different (45.0% and 75.0%, resp.). In another study, Holinka et al. [37]. compared the results of sonication culture to the conventional tissue culture in 60 consecutive patients with loosening of the prostheses or implants. The sensitivity of sonication fluid culture was 83.3%, of single positive tissue culture was 72.2% and 61.1% when two or more cultures yielded the same microorganism. In patients receiving antibiotic therapy, the sensitivity was 65.9%, 57.5%, and 42.5%, respectively. Pathogens detected in a single tissue culture as well as in sonication culture yielded a significantly higher rate of prosthetic infection than conventional tissue culture alone (*P* = 0.008), even in patients receiving continuous antibiotic therapy before explanation (*P* = 0.016). Consequently, sonication seems to be superior to conventional culture.

#### 7.2.2. Specific and Broad Range PCR (Polymerase Chain Reaction)

The development of nucleic amplification techniques appears promising, and studies have shown their ability to detect unknown and fastidious pathogens. Most commonly, specific or broad-range (16 rDNA) PCR was applied to synovial or periprosthetic tissue [4, 35, 38–41]. Broad range PCR of tissue cultures of patients with PJI showed a sensitivity of 50 to 86% [38–41].

However, the performance of PCR in the diagnosis of PJI can be improved by multiplex or specific PCR of sonication fluid from removed implants [34, 35, 42]. In a recent study conducted by Piper et al. [42]., the sensitivity of specific PCR for detection of *P. acnes* and *S. aureus* in sonicated fluid were, respectively, 89% and 97%. In another study, Ackermann and colleagues [34] found that the sensitivity of multiplex of sonicated fluid was better than that of sonicated culture (78% versus 62%), especially in patients who had received previous antibiotic therapy (100% versus 42%, *P* < 0.001).

#### 7.2.3. MALDI-TOF MS (Matrix-Assisted Laser Desorption/Ionization and Time of Flight Mass Spectrometry)

Identification of staphylococci (e.g., coagulase negative staphylococci) or other microorganisms using MALDI-TOF MS is straightforward, and the identification accuracy is equivalent to molecular methods [43–45].

#### 7.2.4. Specific Resistance Detection

With additional molecular tests, specific resistance genes, such as genes conferring resistance to methicillin, can be detected. This information is crucial for targeting the antimicrobial therapy in cases of negative cultures or to reduce the duration of the postoperative empiric antibiotic treatment in an attempt to reduce the prescription of glycopeptides or other anti-methicillin-resistant *Staphylococcus aureus* agents by waiting for the definite culture results.

Titecat et al. [46] have evaluated the rapid detection of methicillin-resistant staphylococci (MRS) by Xpert technology directly on intraoperative samples in patients with chronic PJI. This method was compared to conventional culture for 104 clinical specimens performed on 30 patients. The performance of the test expressed in terms of sensitivity, specificity, positive predictive value, and negative predictive value was, respectively, 87.1%, 100%, 100%, and 94.5% for the 104 specimens and 92.3%, 100%, 100%, and 94.4% for the 30 patients. With the rapid detection of MRS, the use of vancomycin was limited for 17 of these 30 patients.

These results confirm the findings of Dubouix-Bourandy et al. [47] established in patients suffering from miscellaneous bone and joint infections (i.e., PJI, spondylodiscitis, and arthritis). In this study, the median total test turnaround time was 72 min for PCR versus 79 h for culture.

#### 7.2.5. Innovative Techniques (Microcalorimetry, Fluorescence In Situ Hybridization)

Microcalorimetry is a promising method for the rapid diagnosis of PJI in measuring the heat production of bacteria in small quantity (1 to 10 CFU/mL). This method has been used for the detection of microorganisms from cerebrospinal fluid and platelets bags [48] and in experimental meningitis [49], and more recently by Clauss et al. [50], fluorescence in situ hybridization (FISH) has emerged as a molecular alternative that is used to detect and localize the presence or absence of specific DNA sequences. It can be applied to environmental samples and is based on phylogenetic markers at 16 or 23S rRNA, that are less influenced by the growth condition [51–53]. These two innovative techniques are not currently used in the routine practice.

## 8. Radiological Examination

### 8.1. Conventional Methods

Differentiating PJI from aseptic loosening is of crucial importance. Computed tomography (CT) and magnetic resonance imaging (MRI) are hampered by artefacts produced by prosthesis devices themselves. CT or X-ray is useful to appreciate the quality of bone and CT to detect abscesses or bone destruction. Combined leukocyte-marrow scintigraphy has been reported to achieve the diagnostic accuracy of 90% or greater. However, combined leukocyte-marrow is time consuming, labor intensive, costly, not widely available, and potentially hazardous because of direct handling of blood product [54–56]. Antigranulocyte scintigraphy with monoclonal antibodies or antibody fragment may be another attractive approach to detect PJI. In a recent meta-analysis included 522 prosthesis in a 13 pooled studies conducted by Pakos et al. [57], the random effects summarized estimates of sensitivity and specificity as 83% and 80%, respectively.

### 8.2. FDG-PET

F-Fluoro-2-deoxyglucose positron emission tomography (FDG-PET) enables visualization of hyperglycolytic inflammatory cells during infection. It may be attractive alternative because it requires only one injection and one scan. However, controversial results have been reported on the diagnosis value of FDG-PET in detecting PJI, and its utility is still debated [54, 56, 58–67].

Kwee et al. [58] performed a meta-analysis of 11 studies concerning 635 prosthesis. Pooled sensitivity and specificity of FDG-PET for detection of hip and knee PJI were 82.1% (CI 95%, 68–90.8) and 86.6% (CI 95%, 79.7–91.4), respectively. Overall specificity in hip PJI was significantly higher than that in knee PJI (89.8% versus 74.8%, *P* = 0.016). The authors explain the lower specificity in knee PJI by the relative limited knowledge about the incidence and pattern of nonspecific FDG uptake around knee prosthesis. Using filtered back projection seems to be better than iterative reconstruction (98.3% versus 82.3%, *P* = 0.023). Pill et al. [59] investigated 89 patients for revision of painful hip prosthesis. Forty-six patients underwent both FDG-PET and combined leukocyte-marrow scintigraphy. They demonstrated comparable specificity (93% and 95.1%, resp.) and a substantially higher sensitivity (95.2% and 50%, resp.). Van acker et al. [60] investigated 21 patients with a painful knee prosthesis. Sensitivity and specificity were 100% and 73%, respectively, for FDG-PET and 100% and 93% for combined leukocyte-marrow scintigraphy. These results should be interpreted with caution. The overall limitations are the generation of artefacts, characterized by artificial FDG uptake adjacent to prosthesis, and the delay of implantation of infected prosthesis. Nonspecific FDG uptake may be also in healing tissues, up to 6 months after prosthesis implantation, after bone fractures or atherosclerotic lesions [61–64]. The use of standardized uptake value (SUV) has not been yet validated in inflammation and infection as it is in oncology. Therefore, calculation of the SUV should be used with caution in this field.

Recently, Glaudemans et al. [56] proposed an algorithm decision for the management of patients with PJIs with a different pathway depending on the age of prosthesis and the probability of infection. The authors suggested that three-phase bone scan or FDG-PET should be performed in patients with suspected PJI if the hip and knee prostheses have been implanted for more than 2 and 5 years, respectively. In case of positivity, in a patient with a chronic infection, antigranulocyte MoAb is indicated. In cases of acute infection, the choice is three-phase WBC scan. This nucleic exam has been also recommended by the authors for patients with suspected infection if the age of hip and knee prosthesis is inferior to 2 and 5 years, respectively. Further studies are required to compare the diagnostic performance of coupled leukocyte-marrow scintigraphy, FDG-PET, three-phase white blood count scan, and antigranulocyte MoAb and to assess which imaging modality is the most cost effective.

## 9. Treatment

### 9.1. Surgical Treatment

The goal of treating PJI is pain-free and functional joint. A multidisciplinary approach with expertise in this field for combined antimicrobial and surgical treatment should be considered in all cases of PJI. There are various surgical approaches: debridement with implant retention, one or two stage replacement, and permanent removal of implant, with various clinical success rates. In cases of contraindicated surgery, suppressive antibiotic treatment may be proposed. The main limitation is the lacking of randomized studies and the heterogeneous definition of PJI. The surgical approach is individual and depends on the patient, the experience of surgeon, and the susceptibility of microorganisms [1, 7, 68].

### 9.2. Debridement with Retention of Prosthesis

Based on experimental animal models and the knowledge of biofilm pathogenesis, debridement with retention of implant appears to be a reasonable solution for patients if (i) the duration of their symptoms is less than 3 weeks, (ii) the infected implant is stable and not aged for more than four weeks, and (iii) the pathogens are susceptible to antimicrobial agents with a good activity in biofilm and a good bone penetration (e.g., rifampin for staphylococci and fluoroquinolones for gram-negative bacilli). The overall clinical success rates for these selected patients vary from 70 to 90% [69–73].

### 9.3. Recent Advances in One- or Two-Stage Replacement Procedures

The prerequisites for the two-stage replacement are adequate bone stock and minimal comorbidities to allow multiple surgical procedures. The one-stage replacement means that the infected prosthesis, excision of all cement, are removed and a new prosthesis is delivered in the same operative time. Some surgeons use antibiotic impregnated cement to fix the new prosthesis. Whereas one-stage replacement appears to be attractive because it allows earlier mobility, it may expose the patient to the risk of persistent infection. One-stage replacement is usually proposed to patients with good health condition, no sinus tract, and who are infected by susceptible pathogens. The two-stage exchange means that all the infected implants are removed. An antibiotic-loaded spacer is placed in the area to fill the cavity and to deliver large amounts of antibiotics especially gentamicin and/or vancomycin and may allow partial joint mobility. The time between removal of the infected implants and the reimplantation of the prosthesis varies from 2 weeks up to several months, although the actual tendency is to reduce the length of time between removal and reimplantation. Two-stage replacement is indicated for patients with resistant microorganisms including fungal agents and incases of poor soft tissue status. With this method, the success rate is more than 90%, but the rates of cost and morbidity are higher than in one-stage revision due to prolonged hospitalization and immobilization of the patient, who is typically elderly [74, 75]. There is more literature on the utilization of one stage in Europe than in US institutions for hip PJI. This difference may be owing to a low number of patients in United States eligible for this type of procedure.

Resection arthroplasty consists of permanent removal of prosthesis and debridement without reimplantation in case of patients with compromised status and hip prosthesis infection or in some cases of multidrug resistant microorganisms. For knee, arthrodesis or amputation may be considered. Of note, arthroplastic resection does not always result in suppressing the infection, whereas it always results in very poor functionality.

### 9.4. Optimal Antibiotics Regimen for Antibiotic-Loaded Spacers

Polymethylmethacrylate (PMMA) is the standard material used as the delivery vehicle for antibiotics. However, it is surface friendly to biofilm forming bacteria; prolonged exposure to antibiotics at subinhibitory levels may allow mutational resistance to occur. Many biodegradable materials have been evaluated as alternatives including protein-based materials (collagen, fibrin, thrombin, and clotted blood), bone graft, and synthetic polymers (polyanhydride, polylactide, polyglycolide, and polyhydroxybutyrate-cohydroxyvalerate …) under various forms or combinations in orthopedic surgery, but none have been approved by FDA (food drugs administration). During the implantation period of the temporary joint spacer (normally 4–8 weeks in cases of susceptible bacteria), antibiotic therapy is delivered locally. For multidrug resistant bacteria, the optimal delay of reimplantation of new prosthesis is unknown. Two methods of addition of the antibiotic to the cement exist: manually mixing at the time of implantation and industrial mixing by companies which provide premixed antibiotic-loaded cement. The choice of the antibiotic is fundamental. When possible, the choice of antibiotic should be targeted to causative microorganisms, should be chemically and thermally stable, and have a synergistic bactericidal activity when locally combined, without altering the mechanical properties of spacer. Various in vitro studies have been published on the diffusion and elution of antimicrobial agents from cement including aminoglycosides (primarily gentamicin but also tobramycin, amikacin, streptomycin), cephalosporins (including cefazolin, cefotaxime, ceftriaxone, and ceftazidime), vancomycin, and fluconazole. The most commonly mixed antibiotics are gentamicin or tobramycin and vancomycin. With the emergence of bacterial resistance, many authors argue that in cases of acute infections, high doses of antibiotics should be used (i.e., >2 g each 40 g of cement) [76–81]. Given the current spread of vancomycin-intermediate/resistant staphylococci, the use of vancomycin-loaded spacer is questionable. Recently, Kaplan et al. [82] analyzed the effect of antibiotic concentration of daptomycin and tobramycin on cement mechanical properties, in varying concentrations. The authors concluded that 2 g of daptomycin and 3.6 g of tobramycin per 40 g packet of cement should be used to promote daptomycin elution without sacrificing PMMA mechanical properties and confirm the findings of Hall et al. [83]. Cortes et al. [84] have reported the first documented clinical use of daptomycin-impregnated cement in a 79-year-old female with multiple allergy treated from chronic MRSA hip prosthetic infection with success. *P. acnes* was isolated in multiple intraoperative samples. Systemic daptomycin at 6 mg/kg/day and gentamicin were administrated postoperatively for 14 days. The spacer was fashioned by adding 2 g of daptomycin and gentamicin per 40 g packet of cement. A second stage revision surgery was performed at 6 months with no signs of persistent infection. To date, no experimental studies on the use of ceftaroline or telavancin or oritavancin into bone cement have been reported. For multidrug resistant bacilli susceptible to carbapenems or colistin, data with antibiotic-loaded spacer are scarce. Meropenem, imipenem, or colistin are unlikely to affect the mechanical properties of cement and can be used into spacers [80, 85–87]. Papagelopoulos et al. [85] reported the case report of a 75-year-old diabetic woman with an early postoperative infection of a total knee prosthesis due to a multidrug-resistant *Pseudomonas aeruginosa* that was managed successfully with surgical removal of the knee prosthesis, antibiotic impregnated cement and intravenous administration of colistin for 6 weeks, and two-stage reimplantation. For fungal infections, there are few data about amphotericin B, fluconazole, and voriconazole use in spacers [88–92].

## 10. Medical Treatment

Propositions of antimicrobial therapy are summarized in Table 1. Antimicrobial treatment for PJI should be ideally active on both planktonic and sessile bacteria, penetrate into bone and periprosthetic space, and should be well-tolerated. Empirical treatment active on *Staphylococcus *spp. including methicillin-resistance staphylococci and gram-negative bacilli should be performed immediately after the microbiological samples are taken. Recent guidelines about the management of PJI from IDSA guidelines are compared to European recommendations in Table 1 [1, 7, 68]. The recommended duration of treatment of total hip and knee prosthesis infections are 3 and 6 months, respectively [1].

### 10.1. Evidence for Methicillin-Resistance Staphylococci

The optimal antimicrobial therapy is well established for staphylococcal PJI. In the literature, rifampin combinations seem to be the best option for these patients and are widely used in Europe as recommended by some experts and more recently in the US [8, 93, 94].

Conversely to multidrug gram-negative bacilli or fungi-associated PJIs. MRSA does not seem necessarily associated with high failure rates when patients are selected. Senneville et al. [95] reported a retrospective study from 98 patients treated for staphylococcal PJI, according to the algorithm of Zimmerli et al. [1]. After a mean of posttreatment of >3 years, remission of infection was observed in 78.6%. Debridement of PJI was not associated with worse outcome than was their removal (78% versus 100% for one stage, and 84.6% for 2 stage). The treatment failure rate was 19.7% in methicillin-susceptible *Staphylococcus aureus* (MSSA-) infected patients versus 29.4% in MRSA-infected patients (*P* = 0.38). In multivariate analysis, an ASA (American society of anesthesiologists) score ≤ 2, and the use of rifampin-fluoroquinolone combination therapy were 2 independent variables associated with remission.

The rising of MRSA and recent recognition of MRSA with reduced or heterogeneous susceptibility to vancomycin and multidrug-resistant negative bacilli create new therapeutic challenge for the treatment of these infections.

For other gram-positive cocci, limited data are available on the safety and efficacy of daptomycin, ceftaroline, telavancin, or oritavancin in PJIs in adults. Daptomycin and telavancin may be potential alternatives or second-line agents to vancomycin in selected patients. Linezolid, because of an increase in clinically important adverse effects with prolonged use, should be reserved as a second- or third-line agent. Little data exist regarding ceftarolin used in osteomyelitis.

Daptomycin is a cyclic lipopeptide antibiotic with rapid, concentration-dependent bactericidal activity which has been approved for systemic treatment of right-sided endocarditis, complicated skin and soft tissue infections, and bacteremia [96, 97]. Both, in vitro and animal models demonstrated a good activity against logarithmic and stationary phase bacteria and a good penetration into biofilm [98–100]. In experimental animal models [101–103], daptomycin was significantly more effective than vancomycin in eradicating MRSA and MSSA. An in vitro synergy effect has been reported between daptomycin and oxacillin against MRSA, as well as between daptomycin and gentamycin, daptomycin and fosfomycin and more recently daptomycin and trimethoprim against MRSA, daptomycin and rifampin in VRE (vancomycin-resistant *Enterococci*) and MRSA [104–112]. However, the clinical relevance of these findings is still uncertain.

Previous experimental data on daptomycin concentrations in infected bone were discouraging. For instance, in a rabbit model of MRSA osteomyelitis, daptomycin total concentrations were reported to be as low as 0.5 mg/L at 60 min after a single subcutaneous dose of 4 mg/kg. In this model, daptomycin was detectable in infected bone only [101]. With 6 mg/kg/day, the concentration was found to be 4.7 mg/L in metatarsal bones [113]. The experience with daptomycin in bone and joint infections is limited. Falagas et al. [114] performed a systematic review of the available data from patients with osteomyelitis treated with daptomycin until 2007. Three cases series about 53 patients were available. Cure was defined as complete resolution of patient's symptoms and signs. On contrary, persistence of clinical symptoms and/or positive microbiology or imaging tests were considered as failure. Spondylodiscitis, hip and knee infections, septic arthritis, and prosthesis infection were the main infections reported. MRSA was the predominant pathogens. Daptomycin was given intravenously at 6 mg/kg/day. Cure of infection was achieved in 81.1% of the cases. In particular, all patients with osteomyelitis were cured, when daptomycin was administered, and 60% of patients with total joint arthroplasty infection were cured with daptomycin treatment combined to surgical treatment. The range of followup was 4 to 13 months. No adverse effect was reported except one patient with nausea and one patient with mild elevation of CPK (creatine phosphokinase) levels which did not result in discontinuing the treatment. Of note, development of resistance to daptomycin was not seen in any of patients of these cases series except one who had MRSA reduced susceptibility to daptomycin from epidural abscess. More recently, Seaton et al. [115] reported a retrospective series of 220 patients from Eucore, treated from osteomyelitis with 6 mg/kg/day and surgery for 52% of them. The most frequent isolated pathogens were *S. aureus* and coagulase-negative staphylococci. Clinical success was achieved in 75% of patients with a trend towards higher success rates, when the infected implants were removed.

### 10.2. Ceftarolin

Ceftarolin is a novel broad-spectrum cephalosporin with potent activity against MRSA strains, which has been approved for skin and soft tissue infections and pneumonia. In contrast to other classic and new antistaphylococcal drugs, ceftarolin also exhibits antibacterial activity against some common gram-negative pathogens including extended-spectrum beta lactamase (ESBL) microorganisms if associated to NLX104 [116–118]. Data on osteomyelitis are scarce. In an experimental model of osteomyelitis due to MRSA and glycopeptide-intermediate *Staphylococcus aureus* (GISA), Jacqueline et al. [119] compared the antibacterial activity of ceftarolin, linezolid, and vancomycin. Ceftarolin and linezolid were associated with a significantly higher decrease in the bacterial inoculum at day 4 in comparison with vancomycin. More recently, Werth et al. [120–122] demonstrated that ceftarolin increased membrane binding and could enhance the activity of daptomycin against daptomycin-nonsusceptible vancomycin-intermediate *S. aureus*. To date, the penetration into bone has been not yet studied. The exeprience in clinical use is limited to one published case report of a patient treated successfully with ceftarolin for endocarditis and osteomyelitis caused by *S. aureus* resistant to daptomycin [123].

### 10.3. Telavancin

Telavancin is a lipoglycopeptide antibiotic with an excellent bactericidal activity and very low MIC values for MRSA, GISA or vancomycin-intermediate *Staphylococcus aureus* (VISA), and VRE. It is approved for treatment of skin and soft tissue infections and has been successfully used in pneumonia [124]. Yin et al. [125] showed the efficacy of telavancin in the treatment of MRSA osteomyelitis in experimental study with a rabbit model. Smith et al. [126] compared the activity of telavancin and vancomycin against MRSA in planktonic culture and biofilms grown using a range of in vitro models. The planktonic minimum inhibitory concentration (MIC) range for telavancin was lower than that for vancomycin (0.06–0.25 mg/L and 0.5–8 mg/L, resp.). Vancomycin (100 × MIC) killed, on average, 59% of cells in hospital-acquired MRSA biofilms, 44% of cells in community-acquired MRSA biofilms, and 26% of cells in VISA biofilms. Telavancin (100 × MIC) killed, on average, 63%, 49%, and 41% of cells, respectively. The antibiotics showed similar efficacy against MRSA biofilms, but telavancin was more effective against those formed by VISA isolates. As daptomycin, an in vitro synergy has been found between telavancin and rifampin in *Enterococcus faecium* isolates resistant to both linezolid and vancomycin [105]. To date, data on bone penetration of telavancin and on clinical experience for osteomyelitis are still lacking. In a recent paper, Twilla et al. [127] reported 4 patients with MRSA osteomyelitis who failed with vancomycin therapy and who were successfully retreated with telavancin and surgical intervention. One patient had renal impairment directly related to telavancin. The followup of these patients was ranged 1 to 7 months after treatment completion. Telavancin was used at 10 mg/kg/d for 4 to 8 weeks. More recently, Brinkman et al. [128] published a case report of an 18-year-old patient who was treated with telavancin-meropenem-rifampin combination for an osteomyelitis of the foot due to methicillin-resistant *S. aureus* and coagulase negative staphylococci associated with a concomitant skin infection due to *Pseudomonas aeruginosa*. The tolerance and efficacy assessed after a one-year followup were satisfactory.

### 10.4. Oritavancin

Oritavancin is a semisynthetic lipoglycopeptide in clinical development that has activity against MRSA and VRE. Belley et al. [129] reported a synergistic activity of this agent in combination with gentamicin, linezolid, moxifloxacin, or rifampin in killing-time studies against methicillin-susceptible, vancomycin-intermediate, and vancomycin-resistant *S. aureus*. More recently, Vidaillac et al. [130] reported the good activity of oritavancin against *S. aureus* with reduced susceptibility to daptomycin.

### 10.5. Safety and Resistance

Daptomycin is generally well-tolerated, and the main adverse effects are rhabdomyolysis, eosinophilic pneumonia, nausea, vomiting, and paresthesia. The long term tolerability of daptomycin has not yet been reported [96]. Recently, MRSA isolates with increased daptomycin MICs have been reported after treatment with vancomycin; the mechanism of which is unknown [131].

Ceftarolin is well-tolerated as are the other betalactam agents [116]. Resistance to ceftarolin is expected to be limited, as demonstrated in multistep resistance selection studies, but increased MICs have been reported with VRE during serial passage. In contrast, vancomycin-susceptible *E. faecalis* developed spontaneous resistance to ceftarolin [132].

The limitations of use of telavancin are renal dysfunction, propensity to cause QTc prolongation, and alteration of laboratory values of prothrombin time, activated partial thromboplastin time, and international normalized ratio. No resistance or increased MIC has been described [124, 133].

### 10.6. Evidence for Multidrug-Resistant Strains and Fungal Prosthesis Joint Infection

For multidrug-resistant gram-negative bacilli, the challenge is more difficult because data on carbapenems, ceftarolin, and tigecycline diffusion into bone and their potential associations are lacking. Corvec et al. [134] reported the activity of fosfomycin, tigecycline, colistine, and gentamycin against ESBL producing *E. coli* in a foreign-body infection model. The MICs and MBCs in logarithmic and stationary phases were 0.12, 0.12, and 8 *μ*g/mL for fosfomycin, 0.25, 32 and 32 *μ*g/mL for tigecycline, 0.25, 0.5, and 2 *μ*g/mL for colistin, and 2, 8, and 16 *μ*g/mL for gentamycin, respectively. The combination fosfomycin-colistin showed the highest cure rate (67%) in comparison to that of colistin plus tigecycline (50%) or fosfomycin plus gentamycin (42%) or colistin plus gentamycin (33%) or fosfomycin plus tigecycline (25%). The combination of fosfomycin-colistin seems to be a promising treatment option for fluoroquinolone-resistant gram-negative bacilli including ESBL-producing rods.

For fungal infections, clinical experiences have been reported with immunocompromised patients treated with azoles or caspofungin for prosthetic joint infection. In most of the cases, patients were treated with two-stage replacement with a long delay between removal and reimplantation with a global success of 50% [90, 135–141].

According to the recent IDSA guidelines and the European recommendations [1, 7, 68], patients with multidrug-resistant bacilli or fungi associated to prosthetic infection require removal of prosthesis.

### 10.7. Evidence for *Mycobacterium tuberculosis *Prosthesis Joint Infection

Mycobacterium tuberculosis PJI is sporadically described, but its incidence is rising.

Misdiagnosis or delayed diagnosis is common due to disparate clinical presentation or due to lack of tuberculosis history. In the majority of cases, *M. tuberculosis* PJI is caused by reactivation of a dormant nidus infection or by hematogenous dissemination of mycobacterium tuberculosis. The diagnosis depends on cultural and histopathological examination which may reveal acid-fast organisms or caseating granulomas. However, granuloma without caseum is not specific of *Mycobacterium tuberculosis* infection. The diagnosis can be facilitated with PCR method. Optimal treatment remains unclear and usually consists of removal of prosthesis in two stage, in addition to administration of four antituberculosis agents. The duration of antituberculous treatment varies from 12 to 24 months [142–148].

In cases of multidrug-resistant (i.e., to rifampin, isoniazid, fluoroquinolones) or extensively drug-resistant tuberculosis, the recommended regimen is the combination of at least four drugs to which *M. tuberculosis* isolate is likely susceptible and should always include an injectable drug to prevent resistance. However, data on bone penetration of historical antituberculosis agents (i.e., capreomycin, PAS, and ethionamide) are scary [149, 150]. Recently, Caminero et al. [150] described the evidence available of each drug and discussed the basis for recommendations for the treatment of patients with multidrug-resistant or extensively drug-resistant tuberculosis. Several factors should be considered when choosing the appropriate drug: the availability of the drug, the patient's resistance profile, the previous use of drug, and the possibility of toxic adverse events. Linezolid combination may be an interesting option because of its bone penetration and its activity on *Mycobacterium tuberculosis*, but physicians should be aware on long term tolerability (optic neuropathy, peripheral neuropathy, anemia, and thrombocytopenia) [151–153].

## 11. Prevention of PJI

Total joint prosthesis is vulnerable to infection during bloodstream infection episodes. The pre- and perioperative procedures listed under are crucial to limit this risk of bacterial seeding on new prosthesis. A checklist of procedures before and during the prosthetic implantation should be proposed.

### 11.1. MRSA Carriers and Decolonization

nasal decolonization of *S. aureus* with mupirocin has been proven effective in reducing the incidence of bacterial infection in patients undergoing cardiac surgery [154, 155]. In orthopedic surgery, the benefit of such an attitude is still debated [156–159].

However, presurgical screening for *S. aureus* is recommended particularly in hospitals where MRSA-related infections are highly prevalent and in some selected cases (e.g., patients admitted from intensive care or rehabilitation units).

### 11.2. Screening of Latent or Active Infection

Patients with active infection (e.g., pneumonia, urinary tract infection, and skin infection) should be temporarily recused for prosthetic implantation. Despite the absence of any recommendations due to lacking data, latent infection should be screened. In cases of chronic leg ulcer, it seems better to wait for complete wound healing before orthopedic surgery. If this is not feasible, care should be strengthened until the ulcer buds. There are no clear recommendations regarding the management of asymptomatic bacteriuria diagnosed prior to major joint replacement surgery. However, the current literature supports treating with a course of antibiotics. A strategy of treating asymptomatic patients who have urine leukocyte counts greater than 10^5^ CFU/mL with a perioperative course of antibiotics and proceeding with surgery seems to be reasonable. In cases of symptomatic bacteriuria (cystitis) or pyelonephritis, treatment and reprogramming of surgery should be considered [160–165]. For dental procedures, it is usually thought that prophylactic antibiotics are necessary for patients with joint replacements who undergo any dental procedures in an attempt to avoid bacteremia and hematogenous seeding of the implant. In the literature, there is no supportive data favoring antimicrobial prophylaxis [166–169]. Recently, Berbari et al. [166] conducted the first case control study that was designed to determine whether dental procedures with or without antibiotic prophylaxis are risk factors for PJI. They found no increased risk of PJI for patients undergoing high risk or low risk dental procedure who were not given antibiotic prophylaxis (OR, 0.8, 95% CI, 0.4–1.6) compared with the risk of patients who were not undergoing a dental procedure (OR, 0.6; 95% CI, 0.4–1.1). Antibiotic prophylaxis in high or low risk dental procedures did not decrease the risk of PJI (OR, 0.9, 95% CI, 0.5–1.6; OR, 1.2, 95% CI, 0.7–2.2 for hip and knee, resp.). In this study, patients with more than one dental visit/year were 30% less likely to develop PJI. Based on these results, French societies of orthopedic surgery and infectious diseases do not recommend the routine antibiotic prophylaxis prior to dental procedure for patients with joint replacements [68, 168]. Conversely, the American Dental Association and the American Academy of Orthopaedic Surgeons stated that antibiotic prophylaxis is not mandatory for routine dental procedures in most patients with joint replacements, but considered that they could be done in those patients with increased risk, including joint replacement within the past two years, previous infection of a joint replacement, inflammatory arthritis, immunocompromised patients, and dental procedure with high risk of infection or bacteremia. The experts proposed an antibiotic prophylaxis depending on having risk of bacterial seeding or not [170]. All emphases on maintaining good oral hygiene and eradicating dental disease in order to decrease the frequency of bacteremia of dental origin.

### 11.3. Procedures in Perioperative Period

Skin preparation is a major part of the prevention of infection in patients undergoing joint replacement and is based on antibioprophylaxis and the use of laminar flow in operative theater. Antibiotic prophylaxis before prosthesis implantation has been proven to be effective in reducing the risk of infection. The goal of antimicrobial prophylaxis is to achieve serum and tissue drug levels that exceed, for the duration of operation, the MICs for microorganisms likely to be encountered during the operation. The timing of antibiotic prophylaxis is considered to be optimal if it is administered between 30 and 60 min before incision. A single dose of an antimicrobial agent is sufficient for most surgical operations. There is no data that support prolonged use of prophylactic antimicrobials, since it is associated with the emergence of resistant bacterial strains. First or second generation cephalosporins are commonly recommended and should be replaced by glycopeptides (vancomycin or teicoplanin) in cases of higher risk of prevalence of MRSA [1, 7, 68]. The place of the new available anti-MRSA agents (daptomycin, linezolid, and tigecycline) are not clear because of lacking data on bone diffusion. Given the risk of induced bacteremia, all catheters (urinary, arterial, or venous catheterization) should be removed when feasible. Herruzo-Cabrera et al. [171] identified four risk factors for the occurrence of urinary tract infection in postoperative period of prosthesis implantation: a preoperative stay more than 4 days, inadequate perioperative prevention measures, central venous catheterization, and urinary catheterization. In incontinent patients, the layers should be avoided, especially in hip joint prosthesis implantation. At least, the use of antibiotic-loaded bone cement for prophylaxis against infection is not indicated for patients not at high risk for infection who are undergoing routine primary or revision joint replacement with cement [76].

## 12. Conclusion

A better understanding of pathogenesis of prosthetic joint infections, such as microbial interaction with the implant, mechanisms of resistance of microbial cells growing in biofilm and research on the surface treatment of the implants and methods of diagnosis have improved during the past decade. However, prosthetic joint infections are still a devastating infection which is underestimated. The main difficulties of treatment are the limitations of development of new drugs for the five next years, the occurring of multidrug-resistance microorganisms, and the lack of clinical studies in patients with multidrug-resistance gram-negative bacilli. Researches on prevention of infection such as surface treatment of implants are needed.

## Figures and Tables

**Table 1:** European recommendations and IDSA guidelines in the management of prosthetic joint infections [1, 7, 68].

Microorganisms	Swiss guidelines (2007)	French guidelines (2008)	IDSA guidelines (2013)
*Staphylococcus aureus *or coagulase negative staphylococci			
Methicillin-susceptible	Rifampin 450 mg/12 h (IV/PO) + flucloxacillin 2 g/6 h (IV) for 2 weeks followed by oral route: rifampin 450 mg/12 h + ciprofloxacin 750 mg/12 h or levofloxacin 750 mg/d or 500 mg/12 h	(Oxacillin or cloxacillin) 100–200 mg/kg/d (IV) or cefazolin 60–80 mg/kg/d (IV) if penicillin allergy + rifampicin 20 mg/kg/d (IV/P.O.) for 2 weeks followed by oral route (i) First-line treatment: rifampicin 20 mg/kg/d + (ofloxacin 400–600 mg/d or pefloxacin 800 mg/d or ciprofloxacin 1500–2000 mg/d or levofloxacin 500–750 mg/d) (ii) Alternatives to first-line treatment: (1) rifampicin 20 mg/kg/d + fusidic acid 1500 mg/d (2) rifampin 20 mg/kg/d + clindamycin 1800–2400 mg/d (if erythromycin-susceptible) (3) ofloxacin 400–600 mg/d or pefloxacin 800 mg/d or ciprofloxacin 1500–2000 mg/d or levofloxacin 500–750 mg/d + fusidic acid 1500 mg/d (4) clindamycin 1800–2400 mg/d (if erythromycin-susceptible) + fusidic acid 1500 mg/d (5) rifampicin 20 mg/kg/d + cotrimoxazole/trimethoprim 3200 mg/640 mg if no alternative possible	Nafcillin, sodium 1.5 to 2 g/ 4 to 6 h (IV) or cefazolin 1 to 2 g/8 h (IV) or ceftriaxone 1 to 2 g/d + rifampin as a companion drug for rifampin-susceptible PJI treated with debridement and retention or 1-stage exchange in text Alternatives: vancomycin 15 mg/kg/12 h (IV) or daptomycin 6 mg/kg/d (IV) or linezolid 600 mg/12 h (IV/PO) + rifampin as a companion drug for rifampin-susceptible PJI treated with debridement and retention or 1-stage exchange

Methicillin-resistant	Rifampin 450 mg/12 h (IV/PO) + vancomycin 1 g/12 h IV for 2 weeks followed by (1) rifampin 450 mg/12 h (PO) + ciprofloxacin 750 mg/12 h (PO) or levofloxacin 750 mg/d or 500 mg/12 h (PO) (2) rifampin 450 mg/12 h (PO) in addition to (i) teicoplanin 400 mg/24 h after loading dose (IV/IM) (ii) or fusidic acid 500 mg/8 h (PO) (iii) or cotrimoxazole/trimethoprim 1 tablet/8 h (PO) (iv) or minocycline 100 mg/12 h (PO)	Vancomycin 40–60 mg/kg/d (continuous) after a loading dose (15 mg/kg) IV or teicoplanin 12 mg/kg/12 h during 3–5 d then followed by 12 mg/kg/d + rifampin 20 mg/kg/d (IV/PO) Alternatives to rifampin: fusidic acid 1500 mg/d (IV/PO) or fosfomycin 150–200 mg/kg (IV) or doxycycline 200 mg/d (PO) or clindamycin 1800–2400 mg/d (if erythromycin-susceptible) + gentamicin for 2 weeks followed by oral route if possible Rifampicin 20 mg/kg/d in addition to (1) fusidic acid 1500 mg/d (2) or clindamycin 1800–2400 mg/d (if erythromycin-susceptible) (3) or cotrimoxazole/trimethoprim 3200 mg/640 mg (4) minocycline 200 mg/d (5) doxycycline 200 mg/d (6) linezolid 1200 mg/d	Vancomycin 15 mg/kg/12 h (IV) + rifampin as a companion drug for rifampin-susceptible PJI treated with debridement and retention or 1-stage exchange Alternatives: daptomycin 6 mg/kg/d (IV) or linezolid 600 mg/12 h (IV/PO) + rifampin as a companion drug for rifampin-susceptible PJI treated with debridement and retention or 1-stage exchange

*Streptococcus *spp.	Penicillin G 5 million units/6 h (IV) or ceftriaxone 2 g/d (IV) for 4 weeks followed by amoxicillin 750–1000 mg/8 h (PO) (except *S. agalactiae*)	Amoxicillin 100–200 mg/kg/d + gentamicin for 2 weeks then followed by amoxicillin or clindamycin 1800–2400 mg/d	Penicillin G 20 to 24 million units/d (IV) or ceftriaxone 2 g/d (IV) alternatives Vancomycin 15 mg/kg/12 h

*Enterococcus* spp. (penicillin. susceptible)	Penicillin G 5million units/6 h (IV) Or ampicillin or amoxicillin 2 g/4–6 h (IV) + aminoglycoside for 2 to 4 weeks followed by amoxicillin 750–1000 mg/8 h (PO) (and *S. agalactiae*)	Amoxicillin 100–200 mg/kg/d + gentamicin for 2 weeks then followed by oral route: amoxicillin 100–200 mg/kg/d + rifampin 20 mg/kg/d if susceptible	Penicillin G 20 to 24 million units/d (IV) or ampicillin sodium 12 g/d (IV) (continuously or in 6 divided doses) Alternatives: vancomycin 15 mg/kg/12 h (IV) or daptomycin 6 mg/kg/d (IV) or linezolid 600 mg/12 h (PO/IV)

*Enterococcus *spp. penicillin-non susceptible	Vancomycin 15 mg/kg/12 h (IV) + aminoglycoside	Vancomycin 40–60 mg/kg/d (continuous) after a loading dose (15 mg/kg) IV or teicoplanin 12 mg/kg/12 h during 3–5 d then followed by 12 mg/kg/d + rifampin if susceptible or gentamicin.	Vancomycin 15 mg/kg/12 h (IV) Alternatives: daptomycin 6 mg/kg/d (IV) or linezolid 600 mg/12 h (PO/IV)

Enterobacteriaceae quinolone susceptible	Ciprofloxacin 750 mg/12 h (PO)	(Cefotaximi 100–150 mg/kg/d or ceftriaxone 30–35 mg/kg/d) (IV) + (ciprofloxacin 1500–2000 mg/d or ofloxacin 400–600 mg/d) (IV/PO) or gentamicin Alternatives: (imipenem 2-3 g/d or meropenem 3–6 g/d) + gentamicin Then followed by oral route if quinolone susceptible: ciprofloxacin 1500–2000 mg/d or ofloxacin 400–600 mg/d)	IV *β*-lactam based on in vitro susceptibilities or ciprofloxacin 750 mg/12 h (PO) For *Enterobacter* spp.: Cefepim 2 g/12 h (IV) or ertapenem 1 g/d (IV) Alternatives: ciprofloxacin 750 mg/12 h (PO) or 400 mg/12 h (IV)

Nonfermenters (e.g., *Pseudomonas *spp.)	Cefepime or ceftazidim2 g/8 h (IV) + aminoglycoside For 2 to 4 weeks followed by: Ciprofloxacin 750 mg/12 h (PO)	(Ceftazidim or cefepim) Or (imipenem 2-3 g/d or meropenem 3–6 g/d or doripenem) + (amikacin or tobramycin) or ciprofloxacin 1500–2000 mg/d or fosfomycin 150–200 mg/kg/d for 2 to 4 weeks then followed by oral route if possible: ciprofloxacin 1500–2000 mg/d	Cefepime 2 g/12 h (IV) + ciprofloxacin 750 mg/12 h (PO) or 400 mg/12 (IV) Alternatives: meropenem 1 g/8 h (IV) +/− aminoglycosides or ciprofloxacin If aminoglycoside in spacer and organism Aminoglycoside- susceptible then double coverage is provided with recommended IV or oral monotherapy ceftazidim 2 g/8 h (IV)

Anaerobes	Clindamycin 600 mg/6 to 8 h (IV) For 2 to 4 weeks followed by clindamycin 300 mg/6 h (PO)	Gram-positive anaerobes (*Peptostreptococcus* spp., *P. acnes*…) Amoxicillin 100–200 mg/kg/d or cefazolin 6–80 mg/kg/d or clindamycin 1,800–2400 mg/d if erythromycin susceptible. Gram-negative anaerobes (Bacteroides* fragilis,* etc.) Clindamycin 1800–2400 mg/d or metronidazole 1500 mg/d or amoxicillin-clavulanic acid 100 mg/kg/d	For *P. acnes* penicillin G 20 million/d (IV) or ceftriaxone 2 g/d (IV) Alternatives: clindamycin 600 to 900 mg/8 h (IV) or 300 to 450 mg/8 h (PO) or vancomycin 15 mg/kg/12 h

Fungus	No recommendation	Liposomal amphotericin B 0.7 to 1 mg/kg or fluconazole 400 to 800 mg/d (if *Candida* sp. is susceptible) or voriconazole 6 mg/kg/12 h (day 1) then followed by 4 mg/kg/12 h (if candida is susceptible, *Aspergillus* sp.)	No recommendation
